# Regulatory Effects of Fyn on Trophoblast Cell Behaviors and Function

**DOI:** 10.1155/2022/6006981

**Published:** 2022-10-25

**Authors:** Qian Liu, Jinli Ding, Qingzhen Xie

**Affiliations:** Center for Reproductive Medicine, Renmin Hospital of Wuhan University, Wuhan, Hubei 430060, China

## Abstract

Fyn has been proven to be involved in various cell behaviors and pathophysiological processes. However, the expression and roles of Fyn in trophoblasts remain unclear. Here, we aimed to evaluate the participation of Fyn in trophoblast behavior and function, and the related mechanisms were briefly explored. Fyn expression in the HTR-8/SVneo, JEG-3, and JAR cell lines was evaluated by immunofluorescence, quantitative real-time PCR and western blotting. Fyn expression in human hydatidiform moles was also determined by immunohistochemistry and western blot. To explore the effects of Fyn, HTR-8/SVneo and JEG-3 cells were transfected with Fyn shRNA or overexpression plasmid or treated with the Fyn activity inhibitor SU6656 or ERK1/2 inhibitor U0126. The migration, proliferation, and apoptosis of trophoblast cells were assessed using transwell assays, flow cytometry, and cell counting kit-8 assays, respectively. The production of primary inflammatory cytokines, HLA-G and active matrix metallopeptidase (MMP) 2/9, and the phosphorylation of ERK1/2 and STAT3 were evaluated by ELISA, western blot, or gelatin zymography. The results showed that Fyn was expressed by trophoblast cells, mainly in the cytoplasm and membrane. Fyn expression and activity levels both increased in order from HTR-8/SVneo and JAR to JEG-3. The overexpression of Fyn promoted the proliferation and migration of trophoblast cells and inhibited their apoptosis, while the opposite effects were observed for Fyn knockdown and inhibition. Fyn regulated inflammatory cytokine production in trophoblast cells by promoting TGF-*β* and IL-4 secretion while inhibiting IFN-*γ* and TNF-*α* secretion. Moreover, HLA-G expression in JEG-3 was positively regulated by Fyn. Fyn also facilitated the expression of active MMP2/9 and the activation of ERK1/2 and STAT3. Besides, it was confirmed that Fyn regulated trophoblast cell activities through ERK1/2 signal pathway by using U0126. Our study first detected the expression of Fyn in trophoblast cells. Fyn played pivotal roles in trophoblast cell behaviors and function, ERK1/2 was one of its targets, and MMP2/9 and STAT3 may also be involved in the regulatory mechanism.

## 1. Introduction

Fyn, as a prominent member of the Src family kinases (SFKs), has been proven to play pivotal roles in cell behaviors [1]. The chemical structure of Fyn is special, including an N-terminal region required for plasma membrane binding, two Src homology (SH) domains (SH2 and SH3) related to protein–protein interactions, and an adenosine triphosphate- (ATP-) binding site and the C-terminal tail form its catalytic domain which is involved in tyrosine phosphorylation. Fyn functions as a key regulator in tumorigenesis and is considered to be a marker of tumor progression and a target of tumor therapy [2]. The roles of Fyn in immunity and inflammatory reactions are also significant. Through various signaling pathways, Fyn participates in the differentiation and development of T cells and B cells [3, 4] and regulates the secretion of inflammatory cytokines, e.g., interferon-gamma (IFN-*γ*), tumor necrosis factor-alpha (TNF-*α*), tumor growth factor-beta (TGF-*β*), and interleukin-4 (IL-4), by T cells, mast cells, natural killer cells, and macrophages [5–7]. Through the mediation of T helper 17 (Th17) cells, Fyn was confirmed as a negative regulator in fetomaternal immune tolerance in our previous study [8].

Through invasion and migration in uterine and fetal-maternal crosstalk, trophoblast cells play a key role in embryo implantation, pregnancy establishment, and maintenance. Its dysfunction often results in the failure of embryo implantation, spontaneous abortion, gestational trophoblastic neoplasia, hydatid mole, preeclampsia, and fetal growth restriction. The regulatory mechanism of trophoblast cell function is complicated by various cells, mediators, and signaling pathways and is still partially understood [9].

The cell behaviors of trophoblast and tumor are mostly similar. While trophoblast cell behaviors are regulated temporally and spatially, exact and dynamic control makes trophoblast cell invasion restricted to the maternal endometrium and the upper third of the myometrium. We previously detected Fyn expression at the fetomaternal interface, and its expression level fluctuated with the progression of pregnancy, which increased during the preimplantation stage and declined after implantation was completed [8]. Thus, we speculate that Fyn is involved in embryo implantation and trophoblast cell biology. In the present work, we evaluated the participation of Fyn in trophoblast cell behaviors and function, and the related mechanisms were briefly explored.

## 2. Materials and Methods

### 2.1. Cell Culture and Treatments

The HTR-8/SVneo, JEG-3, and JAR cell lines were purchased from the American Type Culture Collection and maintained at 37°C with 5% CO_2_. Cells were cultured in RPMI-1640 (11875-093, GIBCO, NY, USA) and MEM (11095-080, GIBCO) supplemented with 10% fetal bovine serum (10091-148, GIBCO) and 1% penicillin–streptomycin solution (15070-063, GIBCO). HTR-8/SVneo and JEG-3 cells were transfected with Fyn shRNA plasmid, Fyn overexpression plasmid, or empty plasmid or treated with Fyn inhibitor (SU6656, HY-B0789, MCE) or ERK1/2 inhibitor (U0126, HY-12031, MCE). The plasmids were established by Beyotime Biotechnology (Shanghai, China). The transfections were performed according to the Lipofectamine 2000 protocol (11668-019, Invitrogen, Paisley, UK). The transfection efficiency was confirmed by RT–PCR and western blot assays.

### 2.2. Human Samples

A total of 10 fresh villus tissues from human complete hydatidiform moles were collected by surgical abortion, as well as 10 villus fresh tissues from normal pregnant women volunteering to terminate pregnancy. The gestational age ranged from 6 to 10 weeks of amenorrhea, and all of the complete hydatidiform mole cases were diagnosed by pathology. All specimens were obtained with the informed consent of the patients and permission of the Ethical Committee of Renmin Hospital of Wuhan University (No. WDRY2018-K037).

### 2.3. Immunofluorescence Staining and Confocal Microscopy

Trophoblast cells were fixed in 4% paraformaldehyde solution for 15 min and blocked in 1% BSA in PBS for 30 min, followed by incubation overnight at 4°C with a primary antibody against Fyn (1 : 1000, Ab125016, Abcam, Cambridge, MA, USA). Afterwards, Cy3-conjugated secondary antibodies (1 : 1000, BA1032, Boster, Wuhan, China) were added for 1 hr at 37°C. The cell nuclei were stained with 4,6-diamino-2-phenyl indole (DAPI) (C1002, Beyotime Biotechnology, Shanghai, China) for 5 min, and the images were captured using a fluorescence microscope (BX53, Olympus, Tokyo, Japan).

### 2.4. Immunohistochemistry

Villus tissues from hydatidiform mole and normal pregnancy fixed with 4% paraformaldehyde were embedded in paraffin and sliced into 3-5 *μ*m sections. Following dewaxing, hydration, and antigen repair, the sections were treated with 3% hydrogen peroxide solution for 15 min to block endogenous peroxidase activity and goat serum for 30 min to suppress nonspecific activity. Primary rabbit anti-Fyn antibody (1 : 100, A18127, ABclonal, Wuhan, Hubei, China) was incubated on sections overnight. Then, the slides were incubated with an HRP-labelled secondary antibody and stained with 3,3′-diaminobenzidine solution. Images were observed by an inverted microscope, and the positive signal was visualized as brown granules.

### 2.5. Quantitative Real-Time PCR (qRT–PCR)

Total RNA was extracted by using TRIzol reagent (15596-026, Ambionn, Austin, Texas, USA) according to the manufacturer's protocol, and the quality was examined by spectrophotometry. The extracted RNA was then reverse transcribed into cDNA by using HiScript Reverse Transcriptase (R101-01/02, Vazyme Biotech Co., Ltd., Nanjing, China). A 20 *μ*L mixture of the synthesized cDNA (100 ng/*μ*L), primer (20 *μ*mol/L), SYBR Green, and ROX reference dye II (Q111-02, Vazyme Biotech Co., Ltd) was used to perform quantitative RT–PCR on the ABI QuantStudio 6 Real Time PCR System (Applied Biosystems, Richmond, CA, USA). All primers used in the present study were designed and checked by using Primer-BLAST (National Center for Biotechnology Information, Bethesda, MD, USA). The primer sequences were as follows: Fyn forward: 5′-GCACGGACAGAAGATGAC-3′; Fyn reverse: 5′-CCAATCACGGATAGAAAGT-3′; GAPDH forward: 5′-TCAAGAAGGTGGTGAAGCAGG-3′; GAPDH reverse: 5′-TCAAAGGTGGAGGAGTGGGT-3′. Fyn mRNA expression levels were determined by the 2^−*ΔΔ*^CT method normalized to GAPDH.

### 2.6. Western Blot

Total protein was extracted with radioimmunoprecipitation assay (RIPA) lysis buffer supplemented with protease and phosphatase inhibitors (P0013B, Beyotime Biotechnology), and the concentrations of total cellular protein were measured using a BCA assay kit (P0010, Beyotime Biotechnology). Total protein samples (40 *μ*g/gel) were separated via 12% SDS/PAGE gel and transferred to PVDF membranes (IPVH00010, Merck Millipore, Germany) by electroblotting. After blocking in TBST with 5% skimmed milk powder for 1 hr at room temperature (RT), the membranes were incubated with primary antibodies, including anti-Fyn (ab125016, 1 : 1000, Abcam, Cambridge, MA, USA), anti-HLA-G (79769, 1 : 1000, Cell Signaling Technology, Danvers, CO, USA), anti-ERK1/2 (ab54230, 1 : 1000, Abcam), anti-p-ERK1/2 (ab201015, 1 : 1000, Abcam), anti-STAT3 (ab68153, 1 : 1500, Abcam), anti-p-STAT3 (9145, 1 : 2000, Cell Signaling Technology), and anti-GAPDH (AB-P-R 001, 1 : 1000, Goodhere Biotechnology, Hangzhou, China), overnight at 4°C. Then, the membranes were washed and incubated with HRP-labelled goat anti-rabbit secondary antibody (BOSTER Biotechnology, Wuhan, China) for 1 hr at RT. The blot signals were detected by the Odyssey infrared imaging system (LI-COR Biosciences) and analysed by Odyssey software (LI-COR Biosciences).

### 2.7. Immunoprecipitation Assay

Total cell lysate protein was mixed with 50 *μ*L Protein A/G PLUS-Agarose beads (P2012, Beyotime Biotechnology) at a final concentration of 10 *μ*g/*μ*L. Then, the mixture was centrifuged at 10,000 rpm for 10 min at 4°C, and the supernatant was incubated with Fyn antibody (30 *μ*g) for 1 hr at RT. Another 30 *μ*L of Protein A/G PLUS-Agarose beads was added to the supernatant and allowed to react at RT for 20 min. The beads were pelleted and washed three times. Fyn kinase activity was assayed with a Universal Tyrosine Kinase Assay Kit according to the manufacturer's protocol (MK410, Takara Bio, Otsu, Japan).

### 2.8. Cell Migration Assay

The migration ability of cells was detected by a Transwell chamber (MCEP24H48, Millipore, Billerica, MA, USA). In total, 6 × 10^4^ cells in serum-free medium were seeded onto the upper chamber of a transwell precoated with Matrigel matrix, and the lower chamber was filled with complete medium. The chamber was incubated for 24 h at 37°C with 5% CO_2_. Then, the penetrated cells were fixed with 70% ethanol and stained with 0.5% crystal violet (C0121, Beyotime). The number of migrated cells was counted by randomly selecting five fields of view at 200x under a light microscope (OLYMPUS, Japan, BX63).

### 2.9. Cell Viability Assay

Cell proliferation was evaluated by a cell counting kit-8 (CCK-8) assay. Cells in the logarithmic growth phase were seeded into 96-well plates at 5.0 × 10^3^ cells/well. Following transfection or reaction with SU6656 for 24 h, ten microliters of CCK-8 solution was added to each well. A negative control with untreated cells and CCK-8 solution and a blank control with only CCK-8 solution were used. After 4 h of incubation, the absorption values at 450 nm were measured by a microplate reader (Flexstation®, Molecular Devices).

### 2.10. Flow Cytometry

Flow cytometry was applied to determine cell apoptosis. Cells in the logarithmic growth phase were seeded into 6-well plates at 5.0 × 10^5^ cells/well. After transfection or reaction with SU6656 for 24 h, cells were harvested with trypsin and resuspended in PBS. Referring to the AnnexinV-APC/7-AAD kit (KGA1026, KeyGEN BioTECH Corp., Ltd., Nanjing, China) instructions, cell apoptosis rates were determined via flow cytometry (Beckmancoulter, CytoFLEX).

### 2.11. Gelatin Zymography

The presence of matrix metallopeptidase 2 (MMP2) and MMP9 in trophoblast cells was detected by gelatin zymography. Total protein extracted from trophoblast cells was resolved under nonreducing conditions in 10% polyacrylamide gels containing 1 mg/mL gelatin (Difco) via electrophoresis. Gels were washed in 2.5% Triton X-100 for 40 min twice to remove SDS and then in TBS for 20 min twice. Afterwards, the gels were incubated for 24 h at 37°C in renaturing buffer (50 mM Tris–HCl, pH 7.6, 5 mM CaCl2, 0.02% Brij-35). Finally, the gels were stained for 3 h with 0.05% Coomassic Brilliant Blue R-250 (Sigma, St. Louis, MO, USA). After destaining, MMPs were detected as clear bands on a uniform blue background. Quantitative analysis was carried out by using a densitometer (Gel Pro Analyser 4.0).

### 2.12. Enzyme-Linked ImmunoSorbent Assay

The levels of cytokines secreted by trophoblast cells, including IFN-*γ* (E-EL-H0101c, assay sensitivity: 18.75 pg/mL), TNF-*α* (E-EL-H2305c, assay sensitivity: 37.50 pg/mL), IL-4 (E-EL-H0101c, assay sensitivity: 18.75 pg/mL), and TGF-*β* (E-EL-0162c, assay sensitivity: 0.10 ng/mL), were determined using ELISA kits according to the manufacturers' instructions (Elabscience, Wuhan, China).

### 2.13. Statistical Analysis

Data are presented as the mean ± SEM. Statistical analyses were performed using GraphPad Prism version 9.0.0 software (GraphPad, San Diego, CA, USA). Analysis of variance, followed by Brown-Forsythe and Welch ANOVA tests, was used for the comparisons between groups. A two-tailed *p* value < 0.05 was considered significant.

## 3. Results

### 3.1. Fyn Expression and Activity Vary in Different Trophoblast Cell Lines

**Figure 1:**
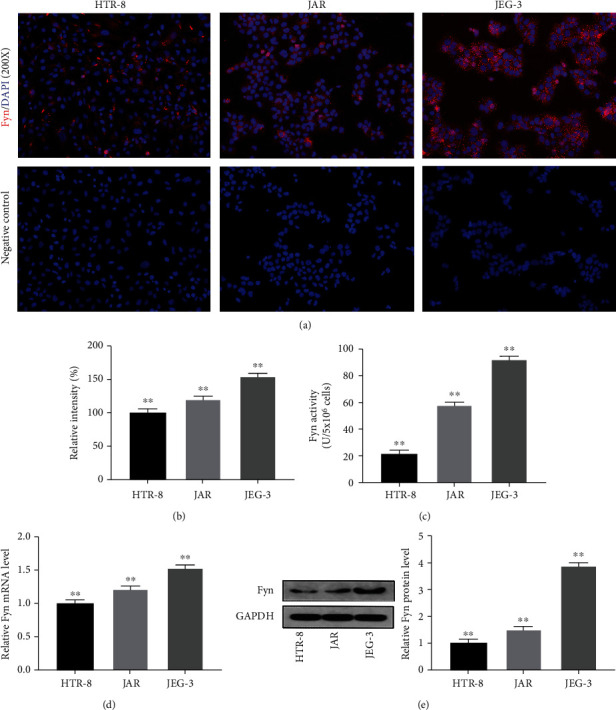
Expression and activity assay of Fyn in trophoblasts. (a) Photomicrographs of Fyn fluorescence by immunofluorescence staining in HTR-8/SVneo, JAR, and JEG-3 cells. (b) Relative intensity of Fyn fluorescence in HTR-8/SVneo, JAR, and JEG-3 cells. (c) Fyn activity levels in three different trophoblast cell lines. (d) Fyn mRNA expression in three different trophoblast cell lines. (e) Fyn protein expression in three different trophoblast cell lines. All experiments were repeated three times. ^∗∗^*p* < 0.01 vs. other groups.

The HTR-8/SVneo, JAR, and JEG-3 cell lines are established in vitro models of placental origin, with distinguishing cell features. Here, we first aimed to determine the expression and activity of Fyn in different trophoblast cell lines. Immunofluorescence staining showed that Fyn was located mainly in the trophoblast cell cytoplasm and membrane and a few in the nucleus. The relative intensity was highest in the JEG-3 cell line and lowest in the HTR-8/SVneo cell line, and the difference between groups was statistically significant (Figures 1(a) and 1(b)). Further quantitative analyses also showed that Fyn mRNA and protein expression and activity levels increased in order from HTR-8/SVneo, JARto JEG-3 (Figures 1(c)–1(e)).

Fyn expression in trophoblasts from normal pregnancy and complete hydatidiform moles was also analysed by immunohistochemistry and western blotting. Hydatidiform mole is the most common type of gestational trophoblastic disease, with aberrant trophoblast cell function. Our results showed a general uptrend in Fyn protein levels in hydatidiform mole compared to normal pregnancy, while the difference was not significant, which might be due to the insufficient sample size (Supplementary Figure 1).

### 3.2. Fyn Affects Trophoblast Cell Migration, Apoptosis, and Proliferation of In Vitro

**Figure 2:**
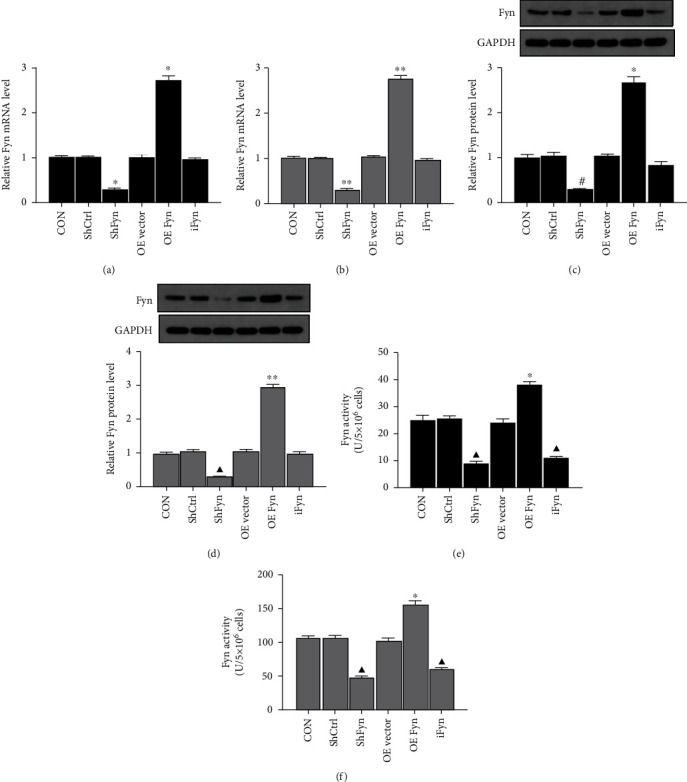
Measurements of Fyn expression and activity under different conditions. (a) The mRNA level of Fyn in HTR-8/SVneo cells. (b) The mRNA level of Fyn in JEG-3 cells. (c) Fyn protein expression in HTR-8/SVneo cells. (d) Fyn protein expression in JEG-3 cells. (e) Fyn activity in HTR-8/SVneo cells. (f) The measurements of Fyn activity in JEG-3. All experiments were repeated three times. ^∗^*p* < 0.05 and^∗∗^*p* < 0.01 vs. all other groups; #*p* < 0.05 vs. the shCtrl and OE vector groups; *^▲^p* < 0.05 vs. the CON, shCtrl, and OE vector groups. iFyn: Fyn inhibitor, SU6656; shCtrl: control shRNA; shFyn: Fyn shRNA; OE vector: overexpression vector; OE Fyn: Fyn-overexpressing vector.

Trophoblast cells (HTR-8/SVneo and JEG-3) were transfected with Fyn shRNA or Fyn-overexpressing vector to modify their gene expression levels. The knockdown and overexpression efficiency was confirmed by qRT–PCR and western blotting; meanwhile, Fyn activity was measured. The effects of SU6656 on Fyn expression and activity were also evaluated. Our results showed that Fyn expression was markedly decreased after gene knockdown but significantly increased after transfection with the Fyn overexpression vector. In addition, Fyn activity was largely modified by gene silencing, overexpression and SU6656 treatment (Figure 2).

**Figure 3:**
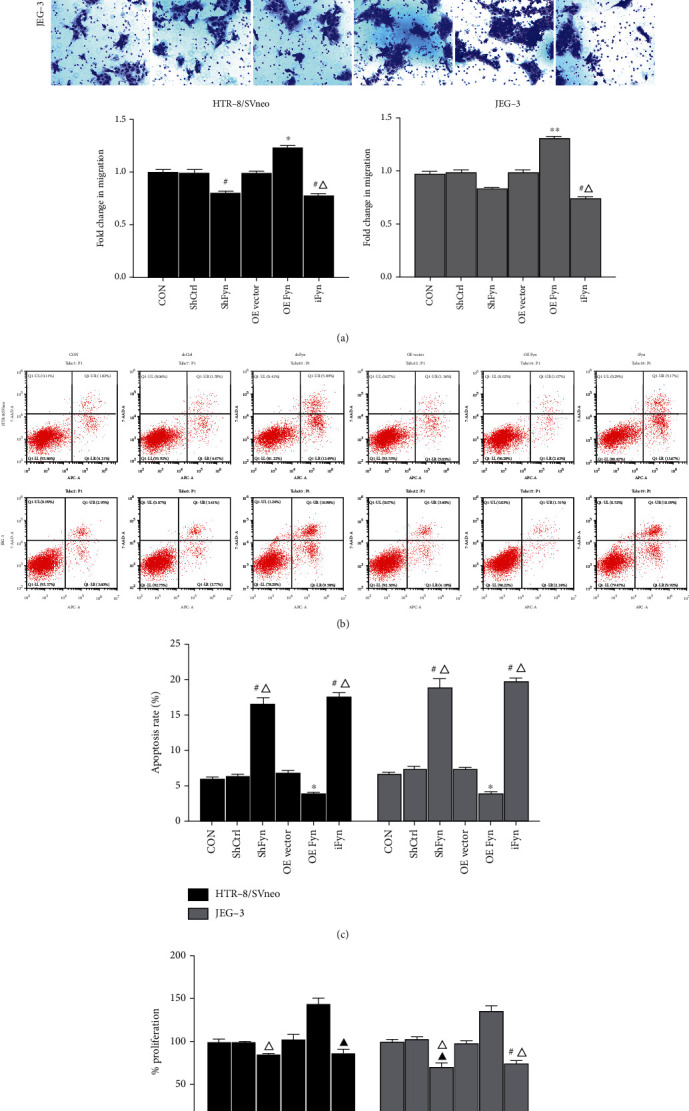
Effects of Fyn on trophoblast cell migration, apoptosis and proliferation. (a) Transwell assays were performed to determine the cell migration ability after Fyn knockdown, overexpression, or treatment with SU6656. (b, c) Cell apoptosis under various conditions was determined by flow cytometry. (d) Cell viability under various conditions was determined by CCK-8 assays. All experiments were repeated three times. ^∗^*p* < 0.05 and^∗∗^*p* < 0.01 vs. all other groups; #*p* < 0.05 vs. the CON, OE vector, and OE Fyn groups; ^▲^*p* < 0.05 vs. the OE Fyn group; ^△^*p* < 0.05 vs. the shCtrl group. shCtrl: control shRNA; shFyn: Fyn shRNA; OE vector: overexpression vector; OE Fyn: Fyn-overexpressing vector; iFyn: Fyn inhibitor, SU6656.

The migration assay demonstrated that Fyn overexpression promoted trophoblast cell migration, while SU6656 restrained cell migration ability in both HTR-8/SVneo and JEG-3 cells; cell migration ability somewhat decreased after the transfection of shFyn, but without significant difference compared with the control shRNA (Figure 3(a)). Next, we assessed the effect of Fyn on the apoptosis of trophoblast cells by flow cytometry (Figure 3(b)). As expected, silencing Fyn by RNA interference and inhibiting Fyn activity with SU6656 both resulted in an apparent increase in trophoblast cell apoptosis. In contrast, the overexpression of Fyn reduced the apoptosis of trophoblast cells (Figure 3(c)). For cell proliferation determined by the CCK-8 assay, Fyn overexpression promoted cell proliferation but had no significant influence compared with the control; SU6656 and shFyn significantly suppressed the proliferation of JEG-3 cells compared with the control group and the control shRNA group, respectively. In HTR-8/SVneo cells, significant differences were found only between the shFyn group and the control group, as well as the Fyn-overexpressing group and the SU6656 group (Figure 3(d)). These combined data suggest that Fyn plays positive roles in the migration, survival, and proliferation of trophoblast cells and that its active state is also of great importance.

### 3.3. Fyn Regulates the Production of Inflammatory Cytokines and HLA-G in Trophoblast Cells

**Figure 4:**
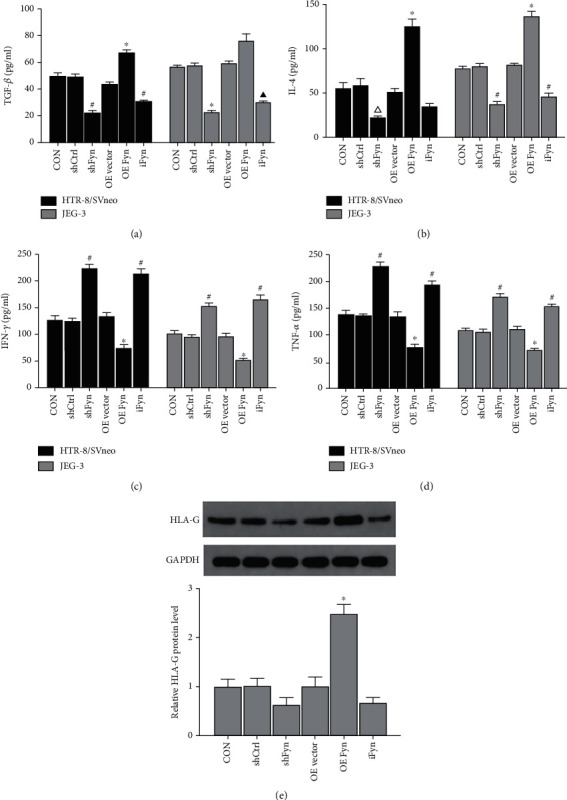
Effects of Fyn on inflammatory cytokine production and HLA-G expression in trophoblast cells. (a–d) Cytokines, including TNF-*α*, IFN-*γ*, IL-4, and TGF-*β*, were measured by ELISA after Fyn knockdown, overexpression, or treatment with SU6656. (e) HLA-G protein expression in JEG-3 cells under various conditions was determined by western blotting. All experiments were repeated three times. ^∗^*p* < 0.05 vs. all other groups; #*p* < 0.05 vs. the CON, shCtrl, OE vector, and OE Fyn groups; *^▲^p* < 0.05 vs. the CON, shCtrl, shFyn, and OE vector groups; ^△^*p* < 0.05 vs. the OE vector and OE Fyn groups. shCtrl: control shRNA; shFyn: Fyn shRNA; OE vector: overexpression vector; OE Fyn: Fyn-overexpressing vector; iFyn: Fyn inhibitor, SU6656.

Cytokines play pivotal roles during the entire pregnancy process, including embryo implantation. Since IFN-*γ*, TNF-*α*, TGF-*β*, and IL-4 are important cytokines in terms of trophoblast cell biology, we detected their content in the culture medium by ELISA (Figures 4(a)–4(d)). Overall, in both HTR-8/SVneo and JEG-3 cells, Fyn overexpression inhibited IFN-*γ* and TNF-*α* expression. In contrast, Fyn knockdown and activity inhibition promoted IFN-*γ* and TNF-*α* expression. TGF-*β*, Fyn overexpression positively regulated its secretion of HTR-8/SVneo cells, while Fyn knockdown and activity inhibition had opposite effects; In JEG-3 cells, Fyn knockdown and activity inhibition markedly suppressed TGF-*β* secretion, while Fyn overexpression did not induce a significant increase in TGF-*β* secretion. A positive relationship between IL-4 secretion and Fyn expression and activity was evident in JEG-3 cells; HTR-8/SVneo cells transfected with the Fyn-overexpressing plasmid secreted more IL-4 than the other groups, while the shFyn plasmid and SU6656 had no significant effects.

Normal trophoblast cells express human leukocyte antigen- (HLA-) I molecules, including HLA-G, HLA-E, HLA-C, and HLA-F, which have a direct immunoregulatory effect and seem to be related to trophoblast cell biological behavior. Here, the protein expression of HLA-G in JEG-3 cells was determined by western blotting (Figure 4(e)). Our results suggested that JEG cells transfected with the Fyn-overexpressing plasmid expressed more HLA-G protein than the other groups; Fyn knockdown and activity inhibition decreased the HLA-G protein level to some degree, but the difference was not statistically significant.

### 3.4. Fyn Expression Increases Active MMP2 and MMP9 Production in Trophoblast Cells

**Figure 5:**
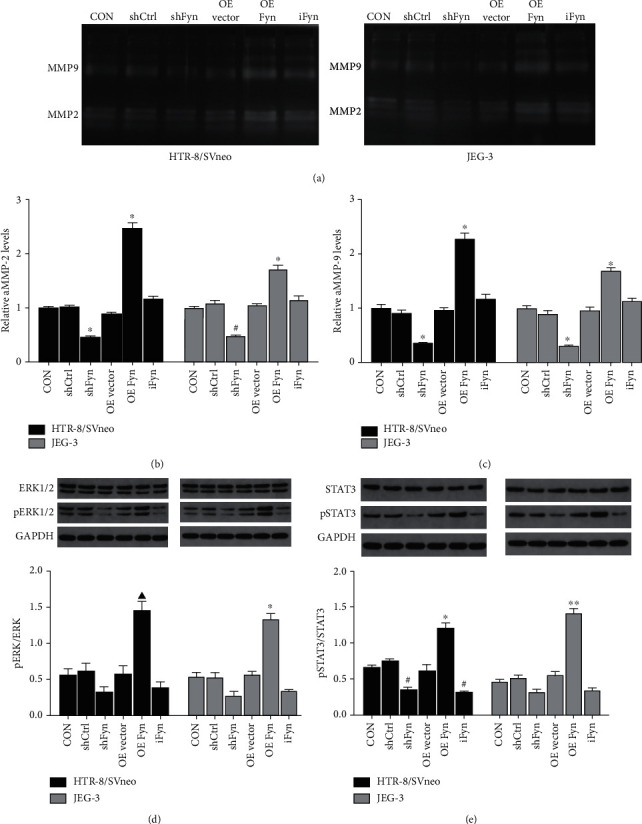
Effects of Fyn on MMP2/9, ERK1/2 and STAT3 activation in trophoblast cells. (a–c) Gelatin zymography was used to measure the production of activated MMP2 and MMP9 in the conditioned medium after Fyn knockdown, overexpression, or treatment with the Fyn inhibitor SU6656. (d, e) The phosphorylation levels of ERK1/2 and STAT3 under various conditions. All experiments were repeated three times. ^∗^*p* < 0.05 and^∗∗^*p* < 0.01 vs. all other groups; #*p* < 0.05 vs. the CON, shCtrl, and OE Fyn groups; *^▲^p* < 0.05 vs. the CON, OE vector, shFyn, and iFyn groups. shCtrl: control shRNA; shFyn: Fyn shRNA; OE vector: overexpression vector; OE Fyn: Fyn-overexpressing vector; iFyn: Fyn inhibitor, SU6656.

Proteolysis of the extracellular matrix (ECM) by matrix metalloproteinases (MMPs) plays a crucial role in the regulation of cell motility. In particular, MMP2 and MMP9 in the placenta could upregulate the migration of trophoblast cells. To determine whether Fyn regulates MMP2 and MMP9 activity in trophoblast cells, active MMP2 and MMP9 levels in the conditioned medium of trophoblast cells with Fyn silence or Fyn-overexpression or Fyn activity inhibition were measured by gelatin zymography (Figures 5(a)–5(c)). We obtained consistent results in HTR-8/SVneo cells and JEG-3 cells. Knockdown of Fyn downregulated the production of active MMP2 and MMP9, and overexpression of Fyn promoted their production. However, 20 *μ*g SU6656 showed no impact. These results suggested that the change in Fyn expression was able to alter active MMP2 and MMP9 production.

### 3.5. Fyn Affects Trophoblast Cell Activities through the ERK1/2 Signaling Pathway

Extracellular signal-regulated kinase (ERK)1/2 and signal transducer and activator of transcription 3 (STAT3) signaling pathways participate in the regulation of trophoblast cell function, and the phosphorylation of ERK1/2 and STAT3 was measured by western blotting in our study. Overexpression of Fyn promoted both ERK1/2 and STAT3 phosphorylation in HTR-8/SVneo and JEG-3 cells. ERK1/2 and STAT3 phosphorylation showed a declining tendency after inhibition of Fyn expression and activity, and a significant decrease was observed in the STAT3 phosphorylation level of HTR-8/SVneo cells (Figures 5(d) and 5(e)).

**Figure 6:**
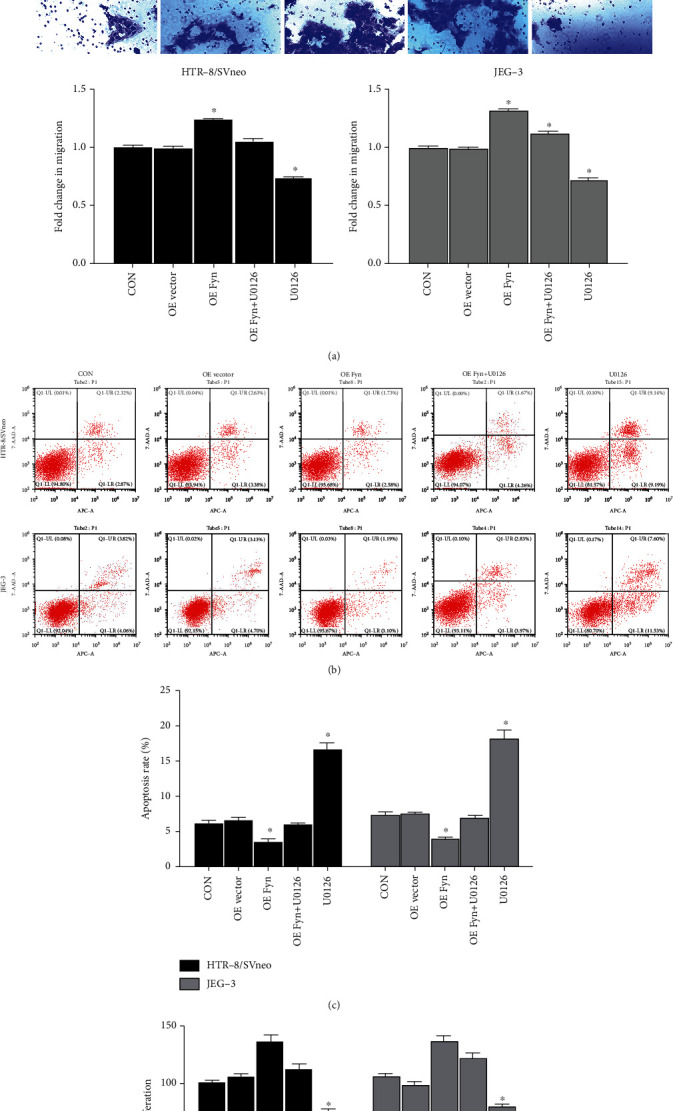
Fyn affects trophoblast cell migration, apoptosis and proliferation through the ERK1/2 signaling pathway. (a) Transwell assays were performed to determine cell migration ability after Fyn overexpression and/or treatment with U0126. (b, c) Cell apoptosis under various conditions was determined by flow cytometry. (d) Cell viability under various conditions was determined by CCK-8 assays. All experiments were repeated three times. ^∗^*p* < 0.05 vs. all other groups. OE vector: overexpression vector; OE Fyn: Fyn-overexpressing vector.

The trophoblast cells were transfected with Fyn-overexpressing vector and/or treated with U0126, an inhibitor of ERK1/2. The migration assay showed that U0126 restrained cell migration ability in both HTR-8/SVneo and JEG-3 cells, and U0126 could partially eliminate the Fyn-induced increase in cell migration ability: the migration of the Fyn-overexpressing+U0126 group decreased compared with that of the Fyn-overexpressing group but still increased compared with that of the U0126 group (Figure 6(a)). The cell apoptosis assay presented similar results (Figures 6(b) and 6(c)). As for cell proliferation determined by CCK-8 assay, because Fyn overexpression had no significant influence on cell proliferation, the proliferation of the Fyn-overexpressing+U0126 group decreased without a statistically significant difference compared with that of the Fyn-overexpressing group. There was also a significant difference between the Fyn-overexpressing+U0126 group and the U0126 group (Figure 6(d)). These data suggested that the ERK1/2 signaling pathway was involved in the roles of Fyn in trophoblast cell activities.

## 4. Discussion

Trophoblast cell growth, proliferation, migration, and invasion are essential for embryo implantation and placentation. Dysfunction in these processes may lead to abortion, fetal growth restriction and death, premature labor, preeclampsia, hydatidiform mole, and so on. The invasion of trophoblast cells into the uterus can be divided into two parts: first, they implant within the decidua to complete embryo implantation; then, extravillous trophoblast cells further invade the inner myometrial layer and spiral artery lumen. Extracellular matrix degradation, angiogenesis, and immune responses are involved, and these processes are regulated by various paracrine and endocrine cytokines, such as integrins, HLA molecules, growth factor receptors, and their ligands [10]. Trophoblast cells and tumor cells are both highly invasive, migratory, and capable of escaping immune system surveillance, and the similarities between tumor cells and trophoblast cells suggest that they may share analogous underlying mechanisms in the regulation of cell function and behaviors.

Fyn, as a pleiotropic nonreceptor tyrosine kinase, plays pivotal roles in a myriad of cellular functions and pathophysiological processes, including cell proliferation, growth, motility, migration and cytokine production, inflammation, and immune reactions. Fyn has been regarded as an oncogene and a potential therapeutic target of different types of tumors, promoting neoplasm cell invasion and metastasis [11]. Nevertheless, far less is known about its function in reproduction. Our previous work found that Fyn expression at the fetomaternal interface is involved in fetomaternal immune tolerance. The expression level of Fyn in uteroplacental units changed with pregnancy progression and peaked at E4.5, implying that Fyn may contribute to implantation and may be involved with trophoblast biology [8].

Fyn expression was first detected in trophoblast cells in our study. The HTR-8/SVneo, JAR, and JEG-3 cell lines are common cellular models of trophoblasts used to understand their function. The HTR-8/SVneo cell line was obtained by immortalization of first-trimester trophoblast cells by transfection with viral vectors. JEG-3 and JAR are both derived from choriocarcinoma cells. These three cell lines show many differences in cell activities and protein expression. HTR-8/SVneo cells have higher invasive and migration ability than JEG-3 cells [12], and JEG-3 cells have been proven to be more metastatic and invasive than JAR cells [13]. We detected Fyn expression in trophoblast cells for the first time, and its expression and activity levels increased in order from HTR-8/SVneo and JAR to JEG-3. Fyn expression was also confirmed in trophoblast cells from normal pregnancy and complete hydatidiform moles. The protein level in complete hydatidiform moles had an increasing tendency, while no significant difference was observed, which may be due to the small sample size. The change in Fyn expression in different trophoblast cells indicates that Fyn is involved in trophoblast cell functions, including but not limited to migration.

To clarify our hypothesis, HTR-8/SVneo cells with the lowest level of Fyn expression and activity and JEG-3 cells with the highest level of Fyn expression and activity were transfected with Fyn shRNA or overexpression plasmid or treated with the Fyn inhibitor SU6656. A series of cell experimental results showed that Fyn exerted positive effects on trophoblast cell proliferation and migration and reduced apoptosis. However, Fyn expression and activity had various degrees of influence on HTR-8/SVneo and JEG-3 cell lines.

Trophoblasts are considered semiallogeneic implants containing foreign genetic material from the father; thus, they might induce maternal immunoreaction. A series of cytokines secreted by trophoblast cells are implicated in maternal-fetal immune balance [14] and simultaneously regulate trophoblast cell migration and growth in an autocrine manner. IFN-*γ*, TNF-*α*, TGF-*β*, and IL-4 are very important for trophoblast cell biology. IFN-*γ* and TNF-*α* are members of the Th1 cytokines, which have been shown to negatively regulate trophoblast cell outgrowth, invasion, and migration and induce apoptosis of human trophoblast cells [15, 16]. TGF-*β* and IL-4 are Th2 cytokines. IL-4 has been reported to promote the proliferation and invasion of trophoblast cell [17]. TGF-*β* mainly protects trophoblasts by modulating maternal-fetal immunity and angiogenesis [18].

Fyn is always characterized as a proinflammatory factor, initiating signaling cascades downstream of immune cells and mediating the production of immune cytokines. The regulatory effects of Fyn on the production of inflammatory cytokines depend on the cell type and the inflammatory state. For example, TNF-*α* production increases in splenocytes but decreases in microglia after Fyn knockout [19, 20]. In our previous study, Fyn was demonstrated to be beneficial for LPS-stimulated IFN-*γ* and TNF-*α* production at the maternal-fetal interface, which included diverse cells, such as immune cells, decidual cells, and trophoblast cells [8]. In this study of trophoblast cells, Fyn expression and activity inhibition downregulated TGF-*β* and IL-4 secretion but accelerated IFN-*γ* and TNF-*α* expression, while Fyn overexpression produced the reverse consequences. These results showed that in addition to its effects on immune cells, Fyn could also affect the immune microenvironment at the maternal-fetal interface by regulating cytokines production by trophoblast cells, which in turn regulate trophoblast biology.

Trophoblast cells selectively express the nonclassical class Ib Ag, including HLA-G, HLA-E and HLA-F, and HLA-C. HLA-G has a direct immunoregulatory effect through binding to receptors expressed on the immune cell surface [21]. Additionally, accumulating evidence suggests that HLA-G is also related to trophoblast cell nonimmune functions, such as syncytiotrophoblast formation, *β*-hCG secretion, and the invasion ability of trophoblast cells [22, 23]. Therefore, it is considered a distinctive feature of invasive EVT, and the only cell line to express endogenous HLA-G is JEG-3. In our study, HLA-G protein expression was found to be elevated after Fyn overexpression. Fyn silence and Fyn inhibitor had no significant impact on Fyn expression, indicating that other regulators were implicated in its expression, playing compensatory roles. These results suggested that HLA-G was another target of Fyn in regulating trophoblast cell function and behavior.

MMPs secreted by trophoblast cells are critical for their migration and invasion ability and successful embryo implantation [24, 25]. MMP2 has been implicated in the remodelling of the ECM to assist trophoblast cell invasion, and a previous study showed that enhancing MMP-2/9 activity could promote trophoblast cell invasion and migration [26]. Remarkably, Fyn was reported to facilitate cell invasion and migration by regulating MMP2 and MMP9 [27, 28], which was in line with our results. MMP-2/9 activity was inhibited by knockdown of Fyn, which in turn was promoted by overexpression of Fyn. However, the Fyn inhibitor had no influence, which may be due to the other compensatory signaling pathways. In this study, SU6656 was used as a Fyn inhibitor [29, 30], and it is a highly selective Src family kinase inhibitor but not specific for Fyn. Thus, these data do not exclude a role for other Src family kinases, including Src, Yes, and Lyn, which may have a different effect from Fyn.

ERK1/2 mainly plays a role in cell proliferation, invasiveness, morphological maintenance, and skeletal remodelling and is closely related to the function of trophoblast cells. A recent study showed that upregulation of MMP-2 and MMP-9 enhanced the invasion ability of trophoblast cells, which was counteracted by the addition of an ERK1/2 inhibitor [31]. In JEG-3, stimulus activated ERK1/2, followed by the phosphorylation of STAT3, and promoted the invasion of cells [32]. In HTR8/SVneo cells, an ERK1/2 inhibitor suppressed proliferation, invasion, and IFN-*γ* secretion [33]. Our study also found that Fyn promoted the phosphorylation of ERK1/2 and STAT3 in trophoblast cells. And the results of experiments using an ERK1/2 inhibitor substantiated that Fyn affects trophoblast cell activities through the ERK1/2 signaling pathway.

## 5. Conclusions

In the present study, we first determined Fyn expression in trophoblast cells, and its expression level and activation status were tightly associated with trophoblast cell behaviors and functions, including proliferation, apoptosis, migration, inflammatory cytokine secretion, and HLA-G expression. ERK1/2 acted as a Fyn target, and STAT3 and MMP2/9 may also be involved in the regulatory mechanism. Fyn functions as a key to trophoblast cell biology and may be applied for the understanding and treatment of implantation failure, spontaneous abortion, and trophoblastic diseases. The roles of Fyn in trophoblast-related pregnancy complications and the specific mechanism still need further research.

## Data Availability

The data that support the findings of this study are available from the author (Dr. Qian Liu) upon reasonable request.

## Supplementary Materials

Supplementary Materials Supplementary Figure 1: Fyn expression in human hydatidiform moles. (A) Immunohistochemical analysis detected the expression of Fyn in villi from complete hydatidiform moles and normal pregnancy (400×). Fyn was located mainly in the trophoblast cytoplasm and membrane. (B) The Fyn protein level in complete hydatidiform moles and normal pregnancy was confirmed by western blot. *n* = 10 samples per group. NP: normal pregnancy; CHM: complete hydatidiform moles.
