# Supplementary figures and images for: Regulatory Effects of Fyn on Trophoblast Cell Behaviors and Function
Source: Biomed Res Int. 2022 Oct 25;2022:6006981. doi: 10.1155/2022/6006981 (PMC9626209; doi:10.1155/2022/6006981)

**A****Fyn****negative control****CON**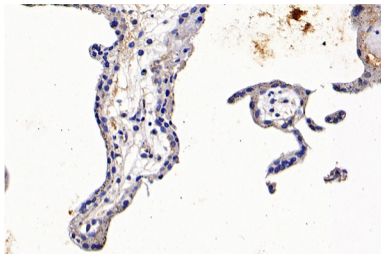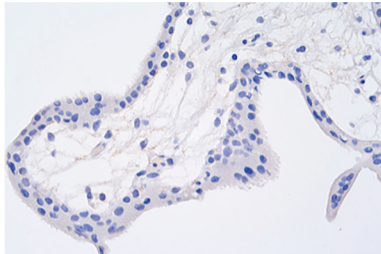**HM**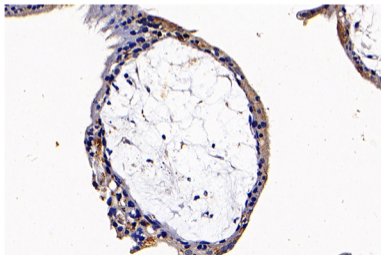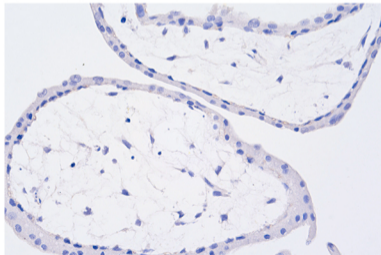**B**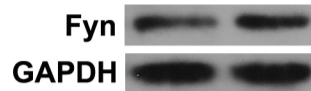**relative Fyn protein level**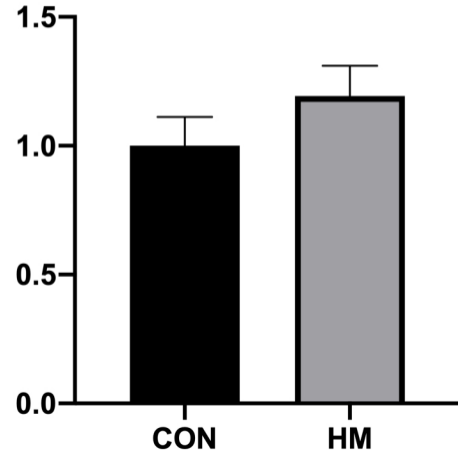

Supplement: Supplementary Materials — Supplementary Figure 1: Fyn expression in human hydatidiform moles. (A) Immunohistochemical analysis detected the expression of Fyn in villi from complete hydatidiform moles and normal pregnancy (400×). Fyn was located mainly in the trophoblast cytoplasm and membrane. (B) The Fyn protein level in complete hydatidiform moles and normal pregnancy was confirmed by western blot. n = 10 samples per group. NP: normal pregnancy; CHM: complete hydatidiform moles. [file 6006981.f1.pdf]
